# Immune Response to Snake Envenoming and Treatment with Antivenom; Complement Activation, Cytokine Production and Mast Cell Degranulation

**DOI:** 10.1371/journal.pntd.0002326

**Published:** 2013-07-25

**Authors:** Shelley F. Stone, Geoffrey K. Isbister, Seyed Shahmy, Fahim Mohamed, Chandana Abeysinghe, Harendra Karunathilake, Ariaranee Ariaratnam, Tamara E. Jacoby-Alner, Claire L. Cotterell, Simon G. A. Brown

**Affiliations:** 1 Centre for Clinical Research in Emergency Medicine, Western Australian Institute for Medical Research and the University of Western Australia, Perth, Western Australia, Australia; 2 Department of Emergency Medicine, Royal Perth Hospital, Perth, Western Australia, Australia; 3 Department of Clinical Toxicology and Pharmacology, Calvary Mater Newcastle and Discipline of Clinical Pharmacology, University of Newcastle, Newcastle, New South Wales, Australia; 4 South Asian Clinical Toxicology Research Collaboration, Peradeniya, Central Province, Sri Lanka; 5 Department of Medicine, Polonnaruwa General Hospital, North Central Province, Polonnaruwa, Sri Lanka; 6 Department of Clinical Medicine, Faculty of Medicine, University of Colombo, Colombo, Sri Lanka; 7 School of Medical Sciences, Faculty of Computer, Health and Science, Edith Cowan University, Perth, Western Australia, Australia; Liverpool School of Tropical Medicine, United Kingdom

## Abstract

**Background:**

Snake bite is one of the most neglected public health issues in poor rural communities worldwide. In addition to the clinical effects of envenoming, treatment with antivenom frequently causes serious adverse reactions, including hypersensitivity reactions (including anaphylaxis) and pyrogenic reactions. We aimed to investigate the immune responses to Sri Lankan snake envenoming (predominantly by Russell's viper) and antivenom treatment.

**Methodology/Principal Findings:**

Plasma concentrations of Interleukin (IL)-6, IL-10, tumor necrosis factor α (TNFα), soluble TNF receptor I (sTNFRI), anaphylatoxins (C3a, C4a, C5a; markers of complement activation), mast cell tryptase (MCT), and histamine were measured in 120 Sri Lankan snakebite victims, both before and after treatment with antivenom. Immune mediator concentrations were correlated with envenoming features and the severity of antivenom-induced reactions including anaphylaxis. Envenoming was associated with complement activation and increased cytokine concentrations prior to antivenom administration, which correlated with non-specific systemic symptoms of envenoming but not with coagulopathy or neurotoxicity. Typical hypersensitivity reactions to antivenom occurred in 77/120 patients (64%), satisfying criteria for a diagnosis of anaphylaxis in 57/120 (48%). Pyrogenic reactions were observed in 32/120 patients (27%). All patients had further elevations in cytokine concentrations, but not complement activation, after the administration of antivenom, whether a reaction was noted to occur or not. Patients with anaphylaxis had significantly elevated concentrations of MCT and histamine.

**Conclusions/Significance:**

We have demonstrated that Sri Lankan snake envenoming is characterized by significant complement activation and release of inflammatory mediators. Antivenom treatment further enhances the release of inflammatory mediators in all patients, with anaphylactic reactions characterised by high levels of mast cell degranulation but not further complement activation. Anaphylaxis is probably triggered by non allergen-specific activation of mast cells and may be related to the quality of available antivenom preparations, as well as a priming effect from the immune response to the venom itself.

## Author Summary

Snakebites cause life-threatening symptoms including uncontrolled bleeding and paralysis. The body's immune responses to snake venom may contribute to the severity of these symptoms but have not been well characterized in humans. Treatment with antivenom is potentially lifesaving, but also carries risk, as severe allergic reactions (anaphylaxis) are common. Anaphylaxis occurs when mast cells, triggered by either allergen-specific antibodies, other immunological mechanisms, or non-immune mechanisms, release mediators that cause skin rashes, shortness of breath and, in severe cases, life-threatening hypotension and/or hypoxia. We have studied 120 snakebite victims in Sri Lanka, both before and after treatment with antivenom. Our results have shown snakebite triggers activation of the complement cascade (an important part of the body's innate immune defence) and production of proinflammatory mediators. In addition, we have demonstrated a quite astonishing level of immune activation after antivenom treatment in virtually every person treated, regardless of whether they had a reaction to the antivenom. Half of the patients treated experienced anaphylaxis, with clear evidence of mast cell activation. Anaphylaxis to antivenom is unlikely to be triggered by allergen-specific antibodies, as patients had not been previously exposed to antivenom, but may be related to the quality of available antivenom preparations, as well as a priming effect from the immune response to the venom itself.

## Introduction

Snake envenoming is a significant medical issue worldwide [1]–[4]. It is a particular problem in South and Southeast Asia, including Sri Lanka, where bites occur from a number of snakes, most importantly Russell's viper (*Daboia russelii*) [5]–[7]. Snake venoms contain an array of proteins, toxins and enzymes that may cause coagulopathy, neurotoxicity, myotoxicity, hypotension and tissue necrosis [1]. In addition to direct toxic effects, *in vitro* studies involving the addition of snake venom to human plasma have shown activation of the complement cascade, with the generation of anaphylatoxins (C3a, C4a, C5a), but these results have not been confirmed *in vivo* in envenomed snakebite victims [8], [9]. Studies of mice injected with various snake venoms have demonstrated release of Interleukin-6 (IL-6), nitric oxide (NO), IL-5, tumor necrosis factor-α (TNFα), IL-4, IL-10, prostaglandins and leukotrienes, with distinct time courses in production post venom exposure for individual mediators [10]–[13]. A small number of studies investigating plasma concentrations of proinflammatory cytokines in envenomed humans have shown elevated concentrations of IL-6, IL-8 and TNFα [14]–[16]. However, these studies were performed on relatively small numbers of patients (n = 14–26) and it remains unknown whether the release of immune mediators contributes to the manifestations of envenoming or simply reflects the degree of tissue damage.

Early systemic reactions to lyophilized equine polyvalent antivenoms, such as those used in Sri Lanka and many other tropical countries, have been reported to occur in up to 75% of patients, with severe reactions (anaphylaxis) in up to 50% of those treated [5]–[7], [17], [18]. Anaphylaxis is classified as either immune-mediated or non-immune-mediated, with immune-mediated further classified as IgE-dependent or non-IgE dependent [19]. IgE-dependent anaphylaxis, where allergen exposure results in crosslinking of allergen-specific IgE bound to the surface of mast cells which subsequently degranulate and release mast cell tryptase (MCT) and histamine, requires prior exposure to the allergen. However, this is unlikely to occur with antivenom because the majority of snakebite victims have not been previously treated. Complement activation by aggregates of immunoglobulins and/or other proteins has therefore been proposed as the principal mechanism underlying early reactions, largely on the basis of *in vitro* studies [1], [20], [21]. The complement system may be activated by envenoming, but no evidence of further complement activation after antivenom treatment has been found in clinical studies [22], [23], and purification to reduce anti-complementary activity does not appear to lower reaction rates [24]. Unlike IgE-dependent anaphylaxis, rigors and fevers are prominent during early antivenom reactions [18], with pyrogens (endotoxins) introduced by poor production processes implicated in these reactions.

We have previously reported elevated serum/plasma concentrations of MCT, histamine, IL-6, IL-10 and the soluble TNF receptor I (sTNFRI) in patients presenting to the Emergency Department with anaphylaxis triggered by foods, insect stings or drugs (predominantly antibiotics and non-steroidal anti-inflammatory drugs (NSAIDs)) [25]. We therefore aimed to investigate both the immune response to envenoming and the mechanisms of adverse reactions to antivenom by measuring markers of complement activation, mast cell degranulation and inflammation.

## Methods

Samples were collected from patients enrolled in a randomized clinical trial comparing two infusion rates of Indian polyvalent antivenom (Bharat Serums and Vaccines Limited, India) for snake envenoming in Sri Lanka which has been described in detail elsewhere [5]. Not all patients in the trial had samples collected. Sample collection was based on logistical considerations (staff availability), not according to whether a reaction occurred.

### Ethics Statement

The study was approved by the Ethical Review Committee, Faculty of Medicine, University of Colombo and was registered with the Sri Lanka Clinical Trials Registry, SLCTR/2007/005. All patients gave written and informed consent to the study. For patients between 14–18 years, written informed consent was obtained from the patient's parents/guardians as well as from the patients themselves.

### Study Patients

Patients were recruited from a secondary referral hospital in Chilaw, Sri Lanka between 21^st^ January 2007 and 31^st^ July 2009. Patients ≥14 years requiring treatment with snake antivenom were eligible for inclusion. Patients administered antivenom prior to recruitment to the study were excluded. After commencing antivenom (10 vials of polyvalent antivenom (Bharat Serums and Vaccines Limited, India) in 500 ml normal saline), patients were observed for four hours for evidence of a systemic hypersensitivity reaction. Blood was collected into EDTA tubes prior to antivenom administration and then 15 minutes, 2 hours, 6 hours, 12 hours and 24 hours after antivenom administration. Samples were immediately centrifuged and the plasma aliquoted and frozen.

### Data Collection

Demographic information, details of the bite, information on snake identification, clinical features of envenoming, treatments and outcomes were recorded. Baseline observations were recorded prior to antivenom administration. The clinical features of early reactions to antivenom were recorded along with blood pressure, heart rate, respiratory rate and pulse oximetry readings.

Clinical envenoming syndrome(s) were defined in each patient as venom induced consumption coagulopathy (INR>1.5), neurotoxicity (ptosis, diplopia, bulbar paralysis, limb weakness and/or respiratory muscle paralysis) or non-specific systemic symptoms (nausea, vomiting, headache, abdominal pain, diarrhoea). The type of snake was based on identification of the snake by the patient or clinician, and confirmed by enzyme immunoassay (EIA) for Russell's viper cases [26].

### Classification of Reactions

Adverse reactions to antivenom were classified as hypersensitivity reactions as we observed “objectively reproducible symptoms or signs, initiated by exposure to a defined stimulus at a dose tolerated by normal subjects” [27]. The use of the term hypersensitivity does not imply previous exposure to antivenom. Systemic hypersensitivity reactions were defined by typical skin manifestations (urticaria/erythema) or National Institute of Allergy and Infectious Diseases/Food Allergy and Anaphylaxis Network (NIAID-FAAN) diagnostic criteria for anaphylaxis [28], which recognise that some reactions may occur in the absence of typical skin features. Our adaptation of this system is presented in Table 1. Because gastrointestinal symptoms were a common effect of envenoming, only new and persistent gastrointestinal symptoms were included in the NIAID-FAAN Definition 2 for the purposes of this study. We also added a further classification of respiratory reactions (Definition 4) to account for the severe respiratory reactions sometimes noted to occur without typical skin features. These reactions were distinguished from those arising from respiratory paralysis because they were not preceded by a descending paralysis and result in primary hypoxia rather than hypoxia secondary to ventilator failure. Reactions were graded as skin only (which does not correspond with a diagnosis of anaphylaxis), moderate anaphylaxis or severe anaphylaxis if hypotension or hypoxia was observed during the course of a hypersensitivity reaction [29]. Pyrogenic reactions (rigors and/or fever) were coded separately and not included in the hypersensitivity reaction grading system.

**Table 1:** Classification of hypersensitivity reactions.

**Skin-only reaction:** Generalised itch, erythema, urticaria, or angioedema without any sentinel features (see below) of other organ involvement
**Anaphylaxis** (based on NIAID-FAAN clinical criteria)
Definition 1: Any skin feature listed above plus at least one sentinel feature from either the respiratory or cardiovascular system (see below)
Definition 2: Sentinel features from two or more organ systems:
1. *Skin* – any of; generalised itch, erythema, urticaria, angioedema
2. *Respiratory* – any of; dyspnea, wheeze, stridor, throat tightness, hypoxemia (cyanosis or SpO_2_ ≤92%)
3. *Cardiovascular* – new onset of absolute hypotension (SBP<90 mmHg)
4. *Gastrointestinal* – new and persistent onset of any of; cramping abdominal pain, nausea, vomiting
Definition 3: Cardiovascular compromise only - new onset of absolute hypotension (SBP<90 mmHg)
Definition 4: Respiratory compromise only, any of; dyspnea, wheeze, stridor, hypoxemia (cyanosis or SpO_2_ ≤92%) not attributable to paralysis
**Anaphylaxis Severity:** Reactions meeting definitions 1–4 above were further classified as “severe” if there was hypoxemia or hypotension; otherwise reactions were designated “moderate” severity.

Treatment of anaphylactic reactions was directed by the attending physician and most commonly involved a combination of parenteral adrenaline (0.25 to 1 mg), corticosteroids (hydrocortisone) and H1-antagonists.

### Laboratory Assays

Plasma concentrations of MCT, histamine, IL-6, IL-10, sTNFRI and TNFα, were measured as described previously [25]. MCT concentrations in plasma were analyzed using the Phadia ImmunoCAP system. MCT results greater than 11.4 ng/ml are considered positive by this system, however we also assessed change in MCT (ΔMCT) with a positive MCT result regarded as a deviation of 2.0 µg/L (ng/ml) between high and low values for each case as measured over time [30]. Histamine concentrations were determined using IBL Histamine ELISA Kits according to the manufacturer's instructions (IBL, Hamburg, Germany). Plasma samples were analyzed for IL-6, IL-10, sTNFRI and TNFα using Flex Sets (BD Biosciences, San Jose, USA). Plasma levels of C3a, C4a and C5a were determined using the Anaphylatoxin CBA Kit from BD Biosciences. We defined a positive result (>upper limit) as a concentration greater than the 99^th^ centile of healthy control samples (n = 34) in our laboratory.

### Statistical Methods

Data are presented as median (interquartile range; IQR). Because of the skewed distributions of mediator concentrations, non-parametric tests were applied to non-transformed (raw) data. The measured baseline and peak mediator concentrations were compared across all groups using the Kruskal-Wallis test and between two groups using the Wilcoxon rank-sum (Mann-Whitney) test. Matched data pairs (i.e. baseline versus peak) were compared using the Wilcoxon signed-rank test. Correlations between baseline and peak mediator concentrations were assessed with Spearman rank correlation and Bonferroni adjustments were applied for multiple simultaneous testing. The semi-parametric lowess smoothing procedure was used to illustrate the relationship between time from antivenom and the concentration of each mediator. Statistical analysis was performed with Stata version 10.1 (StataCorp, College Station, Texas).

## Results

### Patient Demographics and Reaction Characteristics

Of 1004 patients presenting with suspected snakebites during the study period, 247 patients received antivenom. One hundred and ninety eight were randomised to receive antivenom by their allocated infusion rate, and of these, 120 patients had blood samples collected for this study. Baseline characteristics for these patients are presented in Table 2. INR results were available for 112/120 patients, with 111/112 patients presenting with coagulopathy (INR>1.5). The median value for the maximum INR measured was 7.0 (IQR 3.7–13). Signs of neurotoxicity were present in 72/120 (60%) patients and non-specific systemic symptoms were observed in 91/120 (76%) patients. Eighty-six patients bitten by Russell's viper had pre-antivenom venom concentrations measured, with a median of 171(IQR 85–399) ng/ml. Thirty-three patients received a second dose of antivenom a median of 8 (IQR 7–13) hours after the first dose, and six of these patients received a third dose of antivenom.

**Table 2:** Baseline characteristics of 120 envenomed patients.

**Age (yrs)**	38 (27–49)*
**Male, n (%)**	90 (75%)
**Snake, n (%)**	
Russell's Viper	113 (94%)
Cobra	1 (1%)
Krait	1 (1%)
Unknown	5 (4%)
**Time from bite (minutes)**	
To first blood sample	136 (96–209)*
To first antivenom	193 (150–270)*
**Treatment A,** † **n (%)**	64 (53%)

* Median (interquartile range).

† Treatment A is 20 minute infusion rate vs Treatment B 2 hr infusion rate.

Hypersensitivity reactions after the initial dose of antivenom occurred in 77/120 (64%) patients, including 20/120 (17%) with skin-only reactions and 57/120 (48%) with anaphylaxis. All reactions occurred within the first four hours of starting antivenom treatment. Cases of anaphylaxis satisfied Definition 1 in 32/57 (56%), Definition 2 in 11/57 (19%), Definition 3 in 6/57 (11%) and Definition 4 in 8/57 (14%). Severe anaphylaxis was observed in 51/57 (89%) anaphylaxis cases, of which 21/51 (41%) were hypotensive, 18/51 (35%) were hypoxemic and 12/51 (24%) were both hypotensive and hypoxaemic. As reported previously, there was no difference in hypersensitivity reaction rates between the two study arms (i.e. 20 minute vs 2 hour antivenom infusion rate) [5]. Pyrogenic reactions were observed in 32/120 patients (27%), 10/32 (31%) reactions were pyrogenic alone and 22/32 (69%) had a pyrogenic reaction in addition to a hypersensitivity reaction (1 skin-only, 21 anaphylaxis). Pyrogenic reactions were seen more often in patients with anaphylaxis (21/57, 41%) compared to those without (11/63, 17%).

### Pre-antivenom Mediator Concentrations

A comparison between pre-antivenom mediator concentrations in envenomed patients and healthy controls is shown in Table 3. Almost all patients with snake envenoming had evidence of complement activation before receiving antivenom, with plasma concentrations of C3a elevated above the 99^th^ centile of normal in 91% of patients. Plasma concentrations of IL-6, IL-10, C4a and C5a were also significantly higher in envenomed patients before they received antivenom. Although plasma concentrations of MCT were greater than the 99^th^ centile of our healthy control group in 31% of envenomed patients, only 13/120 (11%) patients had MCT concentrations above 11.4 ng/ml, the upper limit of normal defined by the assay (median 15 (IQR 13.9–28.6). Pre-antivenom concentrations of histamine and sTNFRI were not significantly elevated in envenomed patients compared to healthy controls. The median and interquartile ranges for TNFα were zero for both groups.

**Table 3:** Mediator levels in envenomed patients compared to healthy controls.

Mediator	Healthy controls (n = 34)		Envenomed Patients (n = 120) prior to antivenom		
	Median (IQR)	99^th^ centile*	Median (IQR)	n>99^th^ centile	P†
**MCT (ng/ml)**	3.4 (2.6–4.5)	7.4	6.3 (4.3–8.0)	36 (31%)	<0.001
**Histamine (ng/ml)**	0.6 (0.2–0.7)	1.2	0.3 (0.1–0.5)	4 (3%)	0.066
**IL-6 (pg/mL)**	0 (0–0)	7.4	10.3 (0–26.6)	70 (58%)	<0.001
**IL-10 (pg/mL)**	0 (0–3.3)	8.4	20.7 (6.8–57.7)	85 (71%)	<0.001
**sTNFRI (pg/mL)**	1460 (822–2493)	3773	1766 (994–2872)	14 (12%)	0.229
**C3a (ng/ml)**	42.2 (34.7–51.9)	101	1683 (1103–2674)	106 (91%)	<0.001
**C4a (ng/ml)**	820 (534–1091)	2250	1578 (1034–2834)	38 (32%)	<0.001
**C5a (ng/ml)**	7.9 (5.6–10.4)	18.6	27.3 (11.4–113)	73 (62%)	<0.001

* Calculated from assays conducted in our laboratory on healthy controls.

† P-value is Mann-Whitney Test comparing healthy controls and envenomed patients.

Pre-antivenom mediator concentrations did not correlate with maximum INR or pre-antivenom serum venom concentrations (p>0.5 for all comparisons, Spearman's). Patients with signs of neurotoxicity had similar pre-antivenom plasma concentrations of all mediators when compared to patients without signs of neurotoxicity (p>0.2 for all comparisons, Mann Whitney). Patients with non-specific systemic symptoms of envenoming had significantly higher pre-antivenom levels of IL-6, IL-10 and sTNFRI compared to those without (median serum concentrations of IL-6 [12.6 (IQR 0–31.9) *versus* 6.2 (IQR 0–19.5), p = 0.044], IL-10 [29.6 (IQR 10.7–68.7) *versus* 7.6 (IQR 0–16.5), p<0.001] and sTNFRI [2043 (IQR 1131–3257) *versus* 962 (IQR 603–1546), p<0.001]). There was no significant difference in pre-antivenom mediator levels between patients subsequently having no reaction to antivenom and patients experiencing pyrogenic reactions and/or anaphylaxis.

### Mediator Concentrations after Antivenom

**Figure 1:**
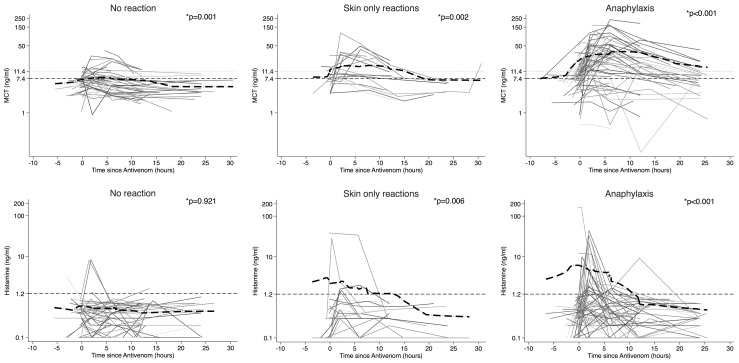
Changes in MCT and histamine concentrations during treatment with antivenom. Lowess curve (thick dash-dot line). Upper limit (99^th^ centile healthy control values from our laboratory) (thin dashed line). Normal limit defined by assay (MCT only) (thin dotted line). * p-value for difference between pre-antivenom concentration and peak concentration during the first four hours post-antivenom (Wilcoxon Signed Rank Test).

**Figure 2:**
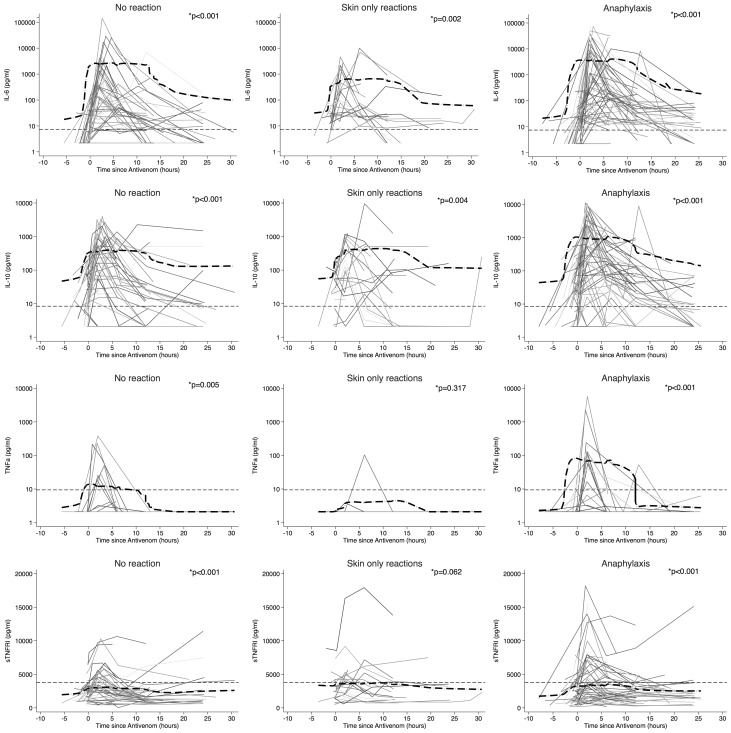
Changes in IL-6, IL-10, TNFα and sTNFRI concentrations during treatment with antivenom. Lowess curve (thick dash-dot line). Upper limit (99^th^ centile healthy control values from our laboratory) (thin dashed line). * p-value for difference between pre-antivenom concentration and peak concentration during the first four hours post-antivenom (Wilcoxon Signed Rank Test).

**Figure 3:**
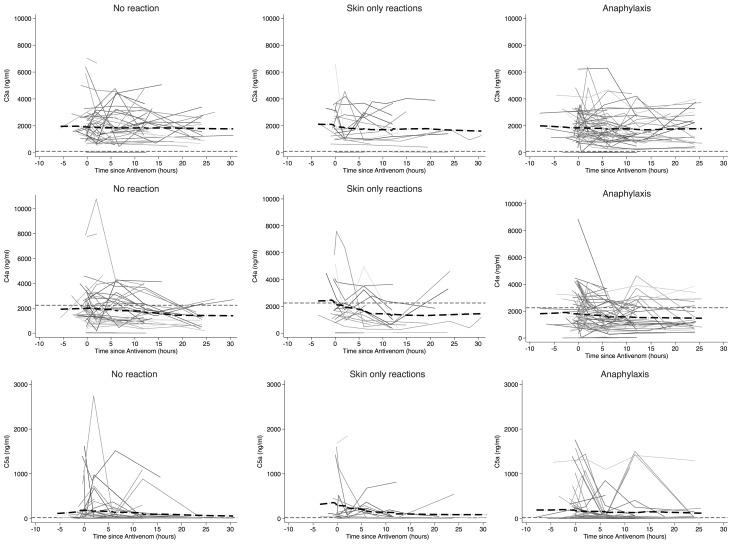
Changes in anaphylatoxin (C3a, C4a and C5a) concentrations during treatment with antivenom. Lowess curve (thick dash-dot line). Upper limit (99^th^ centile healthy control values from our laboratory) (thin dashed line). There were no significant differences between pre-antivenom concentrations and peak concentrations during the first four hours post-antivenom for C3a, C4a or C5a (p>0.13 for all comparisons, Wilcoxon Signed Rank Test).

Changes in serum concentrations of all immune mediators measured before and after treatment with antivenom are illustrated in Figure 1 (MCT and histamine), Figure 2 (IL-6, IL-10, TNFα and sTNFRI) and Figure 3 (C3a, C4a and C5a). As all adverse reactions to the initial dose of antivenom occurred during the first four hours of antivenom treatment, and second and third doses of antivenom were administered at least four hours after the initial dose for all patients, only patients with samples available from the first four hours of antivenom treatment were used for comparisons of baseline (pre-antivenom) and peak mediator concentrations (post-antivenom) by Wilcoxon Signed Rank Test (n = 98). There were significant increases (baseline *versus* peak) in MCT in all patient groups, whereas histamine only increased significantly during skin-only and anaphylactic reactions (Figure 1). Following antivenom administration, peak levels of MCT and histamine were significantly higher in anaphylaxis cases than patients with no reaction and patients with skin-only reactions (Table 4). ΔMCT was positive in 77% of anaphylaxis cases, 56% of skin-only reactions and 31% of patients with no reaction. The proportion of anaphylaxis patients with a positive ΔMCT was significantly higher than patients with no reaction (p<0.001, Fisher's Exact Test). In patients who had anaphylaxis, concentrations of histamine returned to baseline levels between 5–10 hours after the initial dose of antivenom whereas concentrations of MCT remained elevated in some patients for up to 24 hours (Figure 1).

**Table 4:** Peak mediator levels within the first four hours post-antivenom; comparisons between no reaction, skin-only reactions and anaphylaxis.

Mediator	No reaction (n = 35)		Skin only reactions (n = 17)		Anaphylaxis (n = 46)	
	Median (IQR)	n>99^th^ centile*	Median (IQR)	n>99^th^ centile*	Median (IQR)	n>99^th^ centile*
**MCT (ng/ml)** †	7.1 (4.5–9.5)	13 (41%)	9.9 (6.8–28.1)	13 (76%)	24.7 (11.3–49.9)	37 (80%)
**Histamine (ng/ml)** ‡	0.4 (0.2–0.5)	3 (9%)	0.5 (0.3–1.2)	4 (24%)	1.0 (0.4–4.1)	22 (48%)
**IL-6 (pg/mL)**	280 (24.6–2136)	32 (91%)	154 (25.4–1531)	17 (100%)	432 (27.8–9123)	45 (98%)
**IL-10 (pg/mL)**	342 (55.2–937)	32 (91%)	197 (19.1–929)	15 (88%)	606 (71.2–3499)	42 (91%)
**TNFα (pg/mL)**	0 (0–0)	7 (20%)	0 (0–0)	0 (0%)	0 (0–11.9)	12 (26%)
**sTNFRI (pg/mL)**	3476 (2296–5486)	14 (40%)	2296 (1489–5098)	6 (35%)	3362 (1602–7147)	20 (43%)
**C3a (ng/ml)**	2039 (894–2700)	30 (88%)	1442 (956–3032)	13 (81%)	1627 (1149–2463)	41 (89%)
**C4a (ng/ml)**	1516 (933–2857)	13 (38%)	1384 (597–2388)	5 (31%)	1604 (800–2489)	14 (30%)
**C5a (ng/ml)**	26.3 (7.7–197)	18 (53%)	23.2 (14.4–217)	10 (63%)	28.0 (14.4–95.4)	29 (63%)

* Calculated from assays conducted in our laboratory on healthy controls.

† p<0.001 comparing all groups (Kruskal-Wallis), p<0.001 for anaphylaxis versus no reaction, p = 0.050 for anaphylaxis versus skin-only reaction (Mann Whitney).

‡ p<0.001 comparing all groups (Kruskal-Wallis), p<0.001 for anaphylaxis versus no reaction, p = 0.064 for anaphylaxis versus skin-only reaction (Mann Whitney).

Plasma concentrations of IL-6, IL-10, TNFα and sTNFRI all increased significantly after antivenom compared with baseline in all patient groups with the exception of TNFα and sTNFRI in skin-only reactions (Figure 2). However, peak mediator concentrations were not different between patients with no reaction, skin-only reactions and anaphylaxis (Table 4). In patients who had anaphylaxis, concentrations of IL-6 and IL-10 remained elevated for up to 24 hours after the initial dose of antivenom, whereas concentrations of TNFα and sTNFRI returned to baseline levels between 10–15 hours after the initial dose of antivenom (Figure 2).

Figure 3 demonstrates that plasma concentrations of C3a, C4a and C5a were significantly elevated above healthy control levels prior to antivenom, but did not increase significantly in any patient group after administration of antivenom. There were no significant differences in peak concentrations of C3a, C4a and C5a between groups (Table 4).

In agreement with our finding that there was no difference in hypersensitivity reaction rates between the two study arms (i.e. 20 minute vs 2 hour antivenom infusion rate), there was also no significant difference in serum concentrations for any immune mediator measured between the different infusion rates.

### Mediator Concentrations and Pyrogenic Reactions

When all patients were considered together, patients who had pyrogenic reactions to the antivenom had significantly higher peak concentrations of IL-6, IL-10, TNFα and sTNFRI than patients without pyrogenic reactions (data not shown). A confounding factor was that patients with pyrogenic reactions were also more likely to have anaphylaxis. Patients with pyrogenic reactions alone had significantly higher peak levels of IL-6, IL-10 and sTNFRI compared to patients with no reaction (pyrogenic or hypersensitivity) (Table S1). Similarly, patients with both pyrogenic reactions and anaphylaxis had significantly higher peak concentrations of IL-6, IL-10, TNFα and sTNFRI compared to patients with severe anaphylaxis alone.

## Discussion

We found that envenoming by Sri Lankan snakes (predominantly *D. russelii*, Russell's viper) causes significant immune activation with elevated concentrations of MCT, IL-6, IL-10, C3a, C4a and C5a compared to healthy controls, but without clinical evidence of anaphylaxis to the venom. The pre-antivenom concentrations of IL-6, IL-10 and sTNFRI correlated with the non-specific systemic symptoms of envenoming which included nausea, vomiting, headache, abdominal pain and diarrhoea. After treatment with antivenom there were no further changes in C3a, C4a and C5a, but there were rapid increases in IL-6, IL-10, TNFα and sTNFRI concentrations, even when no hypersensitivity reaction to antivenom occurred. These increases were highest in patients who also had pyrogenic reactions. Patients experiencing anaphylaxis also had rapid increases in plasma concentrations of MCT and histamine, with significantly higher increases compared to patients with no reaction to antivenom.

As it would be unethical to have an envenomed control group that did not receive antivenom, an on-going effect of the venom cannot be ruled out as a cause of the increase in cytokine levels after treatment. However, the dramatic nature of the increase, timed with antivenom administration rather than time from bite, and correlations with the severity of hypersensitivity reactions both strongly suggest that the increase reflects an immune response to antivenom in addition to that caused by the envenoming.

Treatment with antivenom is well known to trigger hypersensitivity reactions. Almost half of the patients in this study experienced severe anaphylaxis, characterised by hypoxia and/or hypotension. We observed significant increases in both MCT and histamine concentrations, correlating with the severity of the hypersensitivity reaction. This confirms a role for mast cells and/or basophils in the development of anaphylaxis after antivenom treatment, however, the mechanism of activation by antivenom remains to be elucidated. We observed no increase in C5a, C4a or C3a after treatment with antivenom, with plasma levels remaining steady or declining in the majority of patients post-antivenom. This contradicts previous theories, based on *in vitro* studies, of mast cell degranulation during anaphylaxis to snake antivenoms being caused by complement activation and subsequent binding of anaphylatoxins to their receptors on mast cells [1], [20], [21].

Therefore, the mechanism of mast cell activation during hypersensitivity reactions to antivenom remains to be elucidated. It is highly unlikely that the reactions are allergen-specific IgG or IgE-mediated reactions, because patients rarely have the prior exposure to antivenom that is required to generate allergen-specific antibodies. Repeat exposure to antivenom is very rare in most parts of the world where snake bites are due to unintentional encounters with snakes, unlike developed countries where snake handlers often have prior exposure [31]. Interestingly, reactions to snake antivenom are not more common in snake handlers, again supporting that reactions are not allergen-specific IgG or IgE-mediated. However, anaphylaxis may be triggered by non-allergen specific cross-linking of IgE bound to the surface of mast cells and basophils. For examples, potato lectin has been shown to activate and degranulate both mast cells and basophils by cross-linking cell bound IgE, which contains 10–12% carbohydrates, through interactions with IgE glycans [32]. A similar mechanism of non-allergen specific IgE-triggered anaphylaxis has been proposed for X-ray contrast media reactions, with the hypothesis that contrast media may bridge adjacent IgE molecules via non-specific attachment to their Fc segments [33]. Similarly, it is possible that antivenoms may contain aggregates of immunoglobulins and/or other proteins that have the ability to crosslink IgE receptors on the surface of mast cells resulting in non-allergen specific mast cell degranulation.

Activation of mast cells via IgG receptors has long been proposed as a mechanism for antivenom-induced anaphylaxis. Studies of cultured human mast cells have demonstrated that IgG aggregates can activate mast cells [34], and histamine is released after IgG-receptor FcγRIIA aggregation [35]. Most of these models have required an antigen-specific response and such a mechanism is unlikely in this setting for the reasons outlined above. However, heat-aggregated IgG_1_ (i.e. without the presence of specific antigen) can activate IFNγ-treated human mast cells and this effect is enhanced in the presence of C3a [36]. It is therefore plausible that the immune response to snake venom, including elevated levels of C3a observed in this study, primes cells to degranulate in response to microaggregates of IgG that may be present in antivenom preparations. This may be more likely in preparations that are reconstituted after lyophilisation, if the antivenom (IgG) does not completely dissolve.

Mast cell activation can also occur via a number of other mechanisms, including engagement of receptors for ligands such as chemokines and cytokines, sphingosine 1-phosphate (S1P) and platelet activating factor (PAF) [37]–[40]. An interesting observation in this study was that *prior* to administration of antivenom there was already evidence of immune activation, including high concentrations of cytokines that are produced during anaphylaxis [25]. A proportion of envenomed patients also had clear evidence of mast cell degranulation prior to treatment with antivenom, with elevated baseline concentrations of MCT above normal range in 31%. Histamine concentrations were increased above normal range in some cases but this did not reach statistical significance, which may reflect poorer sensitivity of this assay for low levels of mast cell activation. In a mouse model, Metz *et al* found that mast cells significantly reduce snake-venom-induced pathology, by releasing carboxypeptidase A and possibly other proteases, which can degrade venom components [41]. This probably reflects an innate response to “dangerous” molecular patterns, reflecting the evolution of mast cells to provide a protective role against a range of noxious environmental toxins including venoms [42]. Thus, snake venom-induced activation of mast cells and the release of potent biologically active mediators has been proposed to contribute to the symptoms of envenoming, including local inflammation, vascular permeability (oedema) and shock [12]. It is possible that mast cell activation and the induction of cytokine and anaphylatoxin production by venom also primes cells to react to antivenom.

Pyrogenic reactions, including shivering and sweating, occurred in 32 patients, 10 with no other features of immediate hypersensitivity reaction based on our criteria (Table 1). Pyrogenic reactions to antivenom are thought to be caused by contamination of the antivenom preparation by endotoxin or lipopolysaccharide (LPS). Bacterial contamination of antivenom may also contribute to the development of anaphylaxis as LPS can directly activate mast cells [43]. Therefore, ensuring sterile manufacturing practises and filtering or heat-treating antivenom preparations may reduce both hypersensitivity and pyrogenic reactions [44].

We have demonstrated that Sri Lankan snake envenoming is characterized by significant complement activation and release of inflammatory mediators. Antivenom treatment then intensifies this immune response, resulting in anaphylaxis characterized by high levels of mast cell degranulation in almost half of cases. It is unlikely antivenom reactions are triggered by allergen-specific IgG or IgE. However, we did not observe an increase in C3a, C4a or C5a after administration of antivenom, suggesting anaphylaxis to antivenom is also not triggered by anaphylatoxins binding to their receptors on mast cells and triggering degranulation as previously proposed. Antivenom reactions may be triggered by immunogolublin or proteins complexes present in the antivenom binding to IgG receptors or non-specifically crosslinking IgE on the surface of mast cells. This effect may be related to the quality of available antivenom preparations, as well as a priming effect from immune responses to the venom itself, including venom-induced mast cell degranulation in some patients.

## Supporting Information

Table S1 **Patients who had pyrogenic reactions had higher plasma concentrations of IL-6, IL-10 and sTNFRI compared to patients without pyrogenic reactions in both patients with anaphylaxis and patients with no reaction to antivenom.** Concentrations of TNFα were also higher in patients with both pyrogenic and anaphylactic reactions to antivenom compared to anaphylaxis alone. * Mediator concentrations significantly higher in patients with pyrogenic reactions (Mann-Whitney). ^†^ Mediator concentrations significantly lower in patients with pyrogenic reactions (Mann-Whitney).
(DOC)
