# Supplementary material for: Immune Response to Snake Envenoming and Treatment with Antivenom; Complement Activation, Cytokine Production and Mast Cell Degranulation
Source: PLoS Negl Trop Dis. 2013 Jul 25;7(7):e2326. doi: 10.1371/journal.pntd.0002326 (PMC3723557; doi:10.1371/journal.pntd.0002326)
Supplement: Table S1 — Patients who had pyrogenic reactions had higher plasma concentrations of IL-6, IL-10 and sTNFRI compared to patients without pyrogenic reactions in both patients with anaphylaxis and patients with no reaction to antivenom. Concentrations of TNFα were also higher in patients with both pyrogenic and anaphylactic reactions to antivenom compared to anaphylaxis alone. * Mediator concentrations significantly higher in patients with pyrogenic reactions (Mann-Whitney). † Mediator concentrations significantly lower in patients with pyrogenic reactions (Mann-Whitney). (DOC) [file pntd.0002326.s001.doc]

**Table S1. Patients who had pyrogenic reactions had higher plasma concentrations of IL-6, IL-10 and sTNFRI compared to patients without pyrogenic reactions in both patients with anaphylaxis and patients with no reaction to antivenom. Concentrations of TNFα** were also higher in patients with both pyrogenic and anaphylactic reactions to antivenom compared to anaphylaxis alone.

| **Mediator** | **No reaction (n=27)**  Median (IQR) | **Pyrogenic reaction alone (n=8)**  Median (IQR) | p | **Anaphylaxis alone (n=30)**  Median (IQR) | **Pyrogenic reaction with anaphylaxis (n=16)**  Median (IQR) | p |
| --- | --- | --- | --- | --- | --- | --- |
| **MCT (ng/ml)** | 7.1 (4.4-10.3) | 6.9 (5.9-7.8) | 0.964 | 24.8 (12-49.3) | 23.9 (6.8-52) | 0.982 |
| **Histamine (ng/ml)** | 0.4 (0.2-0.5) | 0.4 (0.2-0.6) | 0.806 | 1.0 (0.3-6.4) | 1.1 (0.4-3.2) | 0.826 |
| **IL-6 (pg/ml)** | 125 (23.2-651) | 2351 (488-4147) | 0.007* | 180 (24.5-1149) | 9155 (375-20426) | 0.008* |
| **IL-10 (pg/ml)** | 164 (51.5-736) | 913 (298-3023) | 0.041* | 210 (60-1859) | 3660 (725-8700) | 0.003* |
| **TNFa (pg/ml)** | 0 (0-0) | 0 (0-22.7) | 0.285 | 0 (0-0.8) | 3.3 (0-195) | 0.020* |
| **sTNFRI (pg/ml)** | 3104 (1486-5433) | 4617 (3452-8567) | 0.028* | 2623 (1462-6635) | 4717 (3124-9385) | 0.038* |
| **C3a (ng/ml)** | 2146 (695-2753) | 1913 (1055-2424) | 0.935 | 1978 (1285-2913) | 1221 (221-1753) | 0.009^†^ |
| **C4a (ng/ml)** | 1431 (682-2988) | 2268 (1222-2701) | 0.330 | 1851 (1052-2556) | 957 (210-2177) | 0.040^†^ |
| **C5a (ng/ml)** | 16.7 (5.4-163 | 127.5 (37.9-420) | 0.113 | 34.7 (15.2-171) | 20.5 (4.1-35.9) | 0.025^†^ |

* Mediator concentrations significantly higher in patients with pyrogenic reactions (Mann-Whitney)

^†^ Mediator concentrations significantly lower in patients with pyrogenic reactions (Mann-Whitney)
